# Deregulation of the FOXM1 target gene network and its coregulatory partners in oesophageal adenocarcinoma

**DOI:** 10.1186/s12943-015-0339-8

**Published:** 2015-03-26

**Authors:** Elizabeth F Wiseman, Xi Chen, Namshik Han, Aaron Webber, Zongling Ji, Andrew D Sharrocks, Yeng S Ang

**Affiliations:** Faculty of Life Sciences, University of Manchester, Michael Smith Building, Oxford Road, Manchester, M13 9PT UK; Faculty of Medical and Human Sciences, University of Manchester, Oxford Road, Manchester, UK; Present address: The EMBL-European Bioinformatics Institute, Wellcome Trust Genome Campus, Hinxton, Cambridge, CB10 1SD UK; Present address: Gurdon Institute and Department of Pathology, Tennis Court Road, Cambridge, CB2 1QN UK

**Keywords:** FOXM1, MMB complex, G2-M cell cycle phase, Oesophageal adenocarcinoma

## Abstract

**Background:**

Survival rates for oesophageal adenocarcinoma (OAC) remain disappointingly poor and current conventional treatment modalities have minimal impact on long-term survival. This is partly due to a lack of understanding of the molecular changes that occur in this disease. Previous studies have indicated that the transcription factor FOXM1 is commonly upregulated in this cancer type but the impact of this overexpression on gene expression in the context of OAC is largely unknown. FOXM1 does not function alone but works alongside the antagonistically-functioning co-regulatory MMB and DREAM complexes.

**Methods:**

To establish how FOXM1 affects gene expression in OAC we have identified the FOXM1 target gene network in OAC-derived cells using ChIP-seq and determined the expression of both its coregulatory partners and members of this target gene network in OAC by digital transcript counting using the Nanostring gene expression assay.

**Results:**

We find co-upregulation of FOXM1 with its target gene network in OAC. Furthermore, we find changes in the expression of its coregulatory partners, including co-upregulation of *LIN9* and, surprisingly, reduced expression of *LIN54*. Mechanistically, we identify LIN9 as the direct binding partner for FOXM1 in the MMB complex. In the context of OAC, both coregulator (eg *LIN54*) and target gene (eg *UHRF1*) expression levels are predictive of disease stage.

**Conclusions:**

Together our data demonstrate that there are global changes to the FOXM1 regulatory network in OAC and the expression of components of this network help predict cancer prognosis.

**Electronic supplementary material:**

The online version of this article (doi:10.1186/s12943-015-0339-8) contains supplementary material, which is available to authorized users.

## Background

Oesophageal adenocarcinoma (OAC) is becoming increasingly common in the Western world and yet five year survival rates remain low (<10%) [1]. Early detection, through the use of molecular markers would help with disease management, as would the identification of new potential therapeutic targets. However compared to other cancers, our knowledge of the deregulated cellular pathways in OACs is much less developed. More recently, genome/systems-wide approaches have been used to accelerate our understanding of the molecular defects in OACs [2]. For example, microarray studies have identified gene signatures that are of prognostic value [3,4] and recent genome-sequencing studies have uncovered new mutations commonly found in OAC samples [5] and associated with disease progression [6].

Many studies have linked the transcription factor FOXM1 to a broad range of different human cancers (reviewed in [7]), including OAC [8]. Moreover, genomic sequencing approaches have revealed defects in the broader FOXM1 regulatory network in the context of high grade serous ovarian cancer [9]. FOXM1 is a key regulator of periodic gene transcription at the G2-M phase of the cell cycle [7,10] and therefore thought to be linked to the increased proliferative nature of tumours. Consistent with this, part of the FOXM1 regulatory network encoding kinetochore-associated proteins was shown to be coordinately upregulated across a range of cancers [11] and another study identified the FOXM1 target gene *CENPF* as synergistically interacting with FOXM1 to drive prostate cancer malignancy [12]. However, while these findings further emphasise a core role for cell cycle-associated FOXM1 target genes in cancer progression, other studies in gliomas have implicated FOXM1 in promoting the nuclear translocation of β-catenin, resulting in activation of a programme of Wnt target genes [13]. This finding is suggestive of alternative roles for FOXM1 in the context of cancer. Indeed, FOXM1 is recruited to DNA in lymphoblastoid cells by NF-kB [14] and FOXM1 co-operates with ERα in driving gene expression in the context of breast cancer [15].

In this study, we took an unbiased approach using ChIP-seq to identify FOXM1 target genes in OAC-derived cells. Subsequently, we studied the expression of a cohort of novel FOXM1 target genes across OAC-derived patient samples. In addition, we investigated the expression of FOXM1 coregulatory partners from the MMB and DREAM complexes in the same patient samples. FOXM1 works synergistically with the MMB complex to drive cell cycle gene expression whereas the DREAM complex functions in an antagonistic manner on the same target genes [16-18]. Our results reveal widespread co-ordinate deregulated expression of the FOXM1 regulatory network in OAC, including changes in both co-regulators (eg *LIN54*) and target genes (eg *UHRF1*) which have potential diagnostic utility in identifying late-stage cancer.

## Results

### Identification of the FOXM1 cistrome in OAC cells

**Figure 1:**
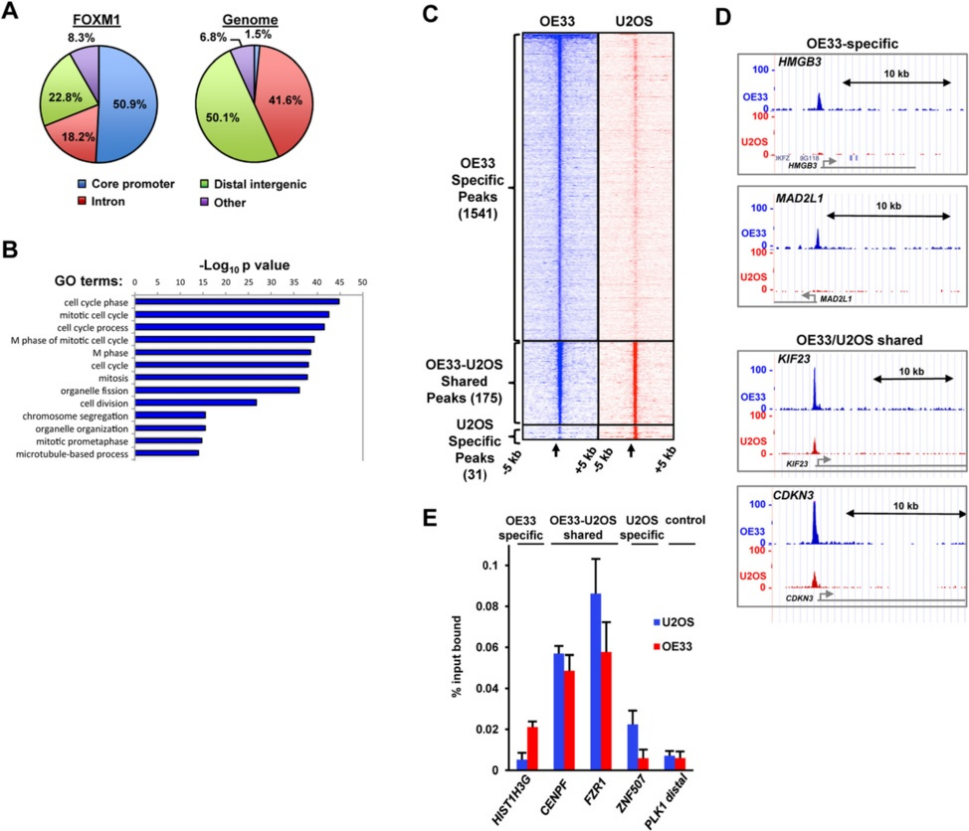
**Identification of the FOXM1 cistrome in oesophageal-derived OE33 cells. (A)** The genomic distribution of the 517 FOXM1 DNA binding regions found in two independent ChIP-seq experiments in OE33 cells (left panel) compared to the background distribution of the same genomic features across the whole genome (right panel). The core promoter corresponds to the 5′ untranslated region (UTR) and DNA sequences 1 kb upstream of the TSS. **(B)** The top 13 enriched GO terms for biological processes identified in the genes associated with the 517 FOXM1 binding regions are shown. Terms are sorted by -log_10_ P-value. **(C)** Heatmaps of the tag density profiles around the peaks identified only in OE33 cells (top panel) or U2OS cells (bottom panel) or in both OE33 and U2OS cells (middle panel) in the OE33 (1,716 peaks; blue) and U2OS (206 peaks; red) ‘combined reads’ datasets. 5 kb upstream and 5 kb downstream of the peak summit (indicated by the arrow) are plotted. **(D)** Screenshots from the UCSC browser showing examples of FOXM1 binding peaks for the indicated genes. Examples of OE33-specific and OE33/U2OS shared binding peaks are shown. **(E)** ChIP-qPCR validation of FOXM1 binding to loci associated with the indicated genes in OE33 (red bars) and U2OS (blue bars) cells. *PLK1* distal is a negative control region not bound by FOXM1. Data are shown as means ± SD (n ≥ 3).

FOXM1 and several of its well established target genes have been shown to be co-overexpressed in OAC [8]. To determine how widespread this co-overexpression is, we first sought to identify all of the direct FOXM1 targets by performing ChIP-seq analysis in the OAC-derived OE33 cell line. In total, 517 high confidence peaks were identified in two independent experiments (Additional file 1: Table S1; for examples see Additional file 2: Figure S1A). We tested a random selection of FOXM1 target regions of varying tag densities by ChIP-qPCR and validated FOXM1 occupancy at 5 out of 6 regions (Additional file 2: Figure S1B). In common with other FOXM1 ChIP-seq studies in different cell types, a large proportion of the binding regions were found in promoter-proximal regions (Figure 1A; [15,16,19]). Moreover gene ontology analysis identified cell cycle-associated GO terms as enriched in FOXM1 target genes, in keeping with its known role in controlling cell cycle events (Figure 1B; reviewed in [20]). Next we compared the FOXM1 binding profile in OE33 cells to previous data derived from osteosarcoma-derived U2OS cells [16]. To provide the biggest possible coverage of FOXM1 binding regions in OE33 cells, the sequencing reads from the two independent ChIP-seq experiments were combined, peaks were recalled and 1716 FOXM1 binding regions were identified (Additional file 1: Table S1). Using this high coverage dataset, we identified 175 binding regions that were commonly occupied in both OE33 and U2OS cells (Figure 1C and D; Additional file 1: Table S1). However, 1541 FOXM1 binding regions were uniquely present in OE33 cells, although a weak tag density profile could be observed in U2OS cells around these peak summits (Figure 1C and D). To validate this differential binding across cell types, we used ChIP-qPCR, and identified the *HIST1H3G* regulatory region as specifically bound by FOXM1 in OE33 cells whereas the opposite was true for the *ZNF507* locus (Figure 1E). Regions bound in both cell types were validated as strongly occupied in OE33 and U2OS cells by ChIP-qPCR (*CENPF* and *FZR1*) (Figure 1E).

**Figure 2:**
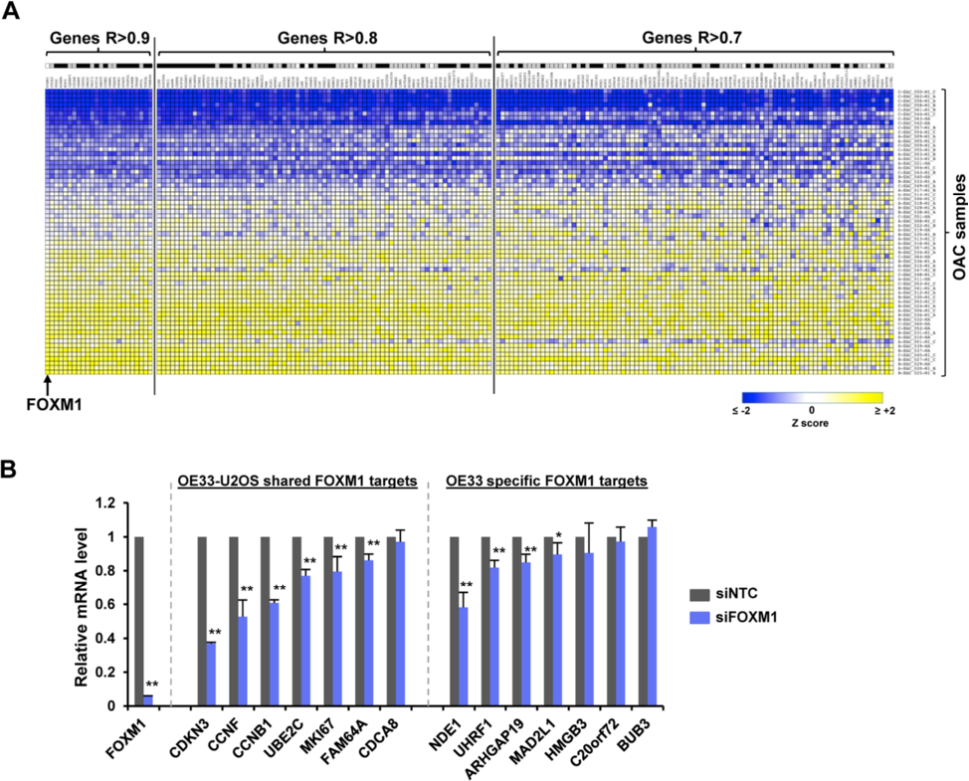
**The role of FOXM1 in regulating its target genes. (A)** Heatmap depicting mRNA expression levels of FOXM1 target genes in oesophageal adenocarcinoma samples. Samples were ranked according to the expression of *FOXM1* across 78 adenocarcinoma samples [4] and genes ranked according to their similarity to FOXM1 expression across all samples (Pearson’s correlation coefficients >0.7 are shown). Dark squares shown above each gene symbol indicate that the gene was in the OE33-U2OS “shared” FOXM1 target gene dataset. **(B)** Nanostring nCounter gene expression analysis of mRNA levels of FOXM1 target genes in OE33 cells following transient transfection with siRNA directed against FOXM1 (siFOXM1, blue bars), for targets shared between OE33 and U2OS cells or specifically found in OE33 cells. The mRNA count was normalised by the geometric mean of *ALAS1*, *GAPDH* and *HMBS* expression. The mean count relative to control cells (siNTC, taken as 1, grey bars) of at least three biological replicates is shown. Error bars indicate the standard deviation. Statistical significance is indicated (P-values: ** <0.01 and * <0.05).

Having established FOXM1 binding, we next wanted to establish FOXM1-dependent gene regulation at its target loci. We reasoned that as FOXM1 is a transcriptional activator, its target genes should be co-upregulated in cancer cells. We therefore compared the expression of *FOXM1* in a microarray study of OACs [4] with that of its high confidence target genes identified by ChIP-seq analysis in OE33 cells. Importantly, 64% of the FOXM1 target genes exhibited highly correlated expression with *FOXM1* across cancer samples (R values >7; Figure 2A). Furthermore, this correlation was maintained in both the OE33-specific and cell type-independently bound FOXM1 target gene subsets, albeit to a greater extent with the latter set of targets. These results are therefore indicative of a role for FOXM1 in regulating a high proportion of its direct target genes in OAC. To further substantiate this activating role for FOXM1, we used the Nanostring nCounter gene expression assay to profile the effect of FOXM1 depletion on the expression of a subset of its target genes in OE33 cells. We focussed on genes whose expression was highly correlated with FOXM1 expression in OAC (R values >7). The majority of the genes commonly occupied by FOXM1 across cell types showed significant reductions in expression upon FOXM1 depletion (Figure 2B; left). The effect on target gene expression from the OE33-specific category was less pronounced but several genes exhibited reduced expression following FOXM1 knockdown, most notably *NDE1* and *UHRF1* (Figure 2B; right).

Together these results therefore identify the binding regions constituting the FOXM1 cistrome in OAC-derived OE33 cells, some of which appear to be preferentially occupied in this cell type compared to U2OS cells. Loss of function experiments demonstrates the importance of FOXM1 for their expression whereas co-expression across OAC cancer samples is suggestive of a role for FOXM1 in driving the expression of a large proportion of its target gene network.

### The expression of FOXM1 target genes and its coregulators in OAC patient biopsies

Although our cross-correlation analysis of FOXM1 expression with that of its target genes in a published microarray study established a close relationship between FOXM1 and the expression of many of its direct target genes, the patient cohort was relatively homogenous, and samples were derived from post-operative samples from patients pre-treated with chemotherapy [4]. We therefore used the Nanostring nCounter gene expression assay to profile the expression of a panel of 49 “direct” FOXM1 targets across our own cohort of patients. 82 clinical samples were analysed: 58 of these were from OAC tissues and the remaining 24 were from normal oesophageal tissues taken from patients with histologically normal oesophageal mucosa and no diagnosis of oesophageal cancer. Basic demographics and, where appropriate, clinical staging and treatment information for the 82 cases analysed is contained in Additional file 3: Table S2. In addition to testing downstream targets, we also profiled the expression of several FOXM1 co-regulatory partner proteins to gain a picture of the broader regulatory circuitry that is operational in OAC. Here we focused on members of the MMB and DREAM complexes which reciprocally co-activate or repress FOXM1 target genes [16,17]. Genes were grouped into those encoding MuvB core complex components (*LIN9*, *LIN37*, *LIN52*, *LIN54*, and *RBBP4)*, DREAM-specific components (*RBL2*, *E2F4* and *TFDP1)* and the MMB-specific component (*MYBL2*).

**Figure 3:**
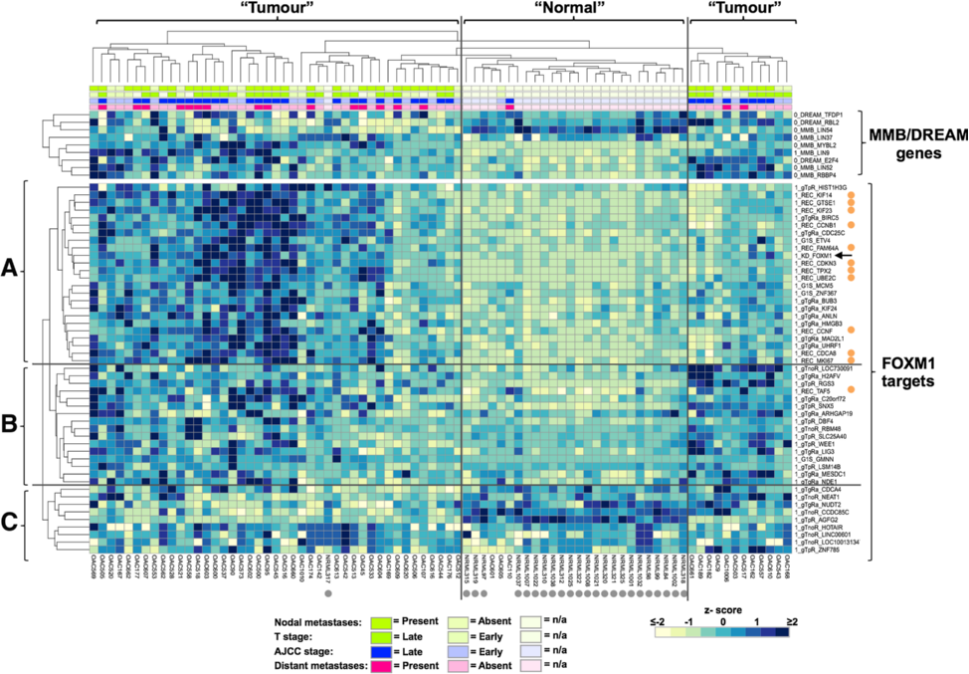
**Heatmap representation of expression of the FOXM1 regulatory network in OAC patient samples.** Heatmap summary of Nanostring nCounter gene expression analysis of 49 direct FOXM1 target genes (bottom panel) or genes encoding members of the MMB and DREAM complex (top panel), in samples from normal and tumour tissues. The expression level of each gene is represented by the z-score of the normalised mRNA level across all samples. The mRNA levels were normalised by the geometric mean of the *GAPDH*, *ALAS1*, *PARPBP*, *HMBS* and *SDHA* internal reference genes. Blue and cream represent high and low expression respectively as indicated by the scale bar. Rows (representing individual genes) and columns (representing individual tissue samples from normal (NRML) and tumour (OAC) tissues) are ordered by unsupervised hierarchical clustering of the 49 FOXM1 target genes. Major clusters of genes are indicated **(A-C)** and samples are broadly categorised into clusters of normal or tumour samples. Genes bound by FOXM1 in both OE33 and U2OS cells are marked by an orange dot. The position of FOXM1 is indicated by the black arrow. Clinical information on overall (AJCC) tumour stage (early/late), T stage (early/late) and presence or absence of nodal or distant metastasis is shown for each tumour sample above the heatmap (top four rows, coloured boxes). Darker coloured boxes represent late T/AJCC stage or presence of nodal/distant metastasis and lighter coloured boxes represent early T/AJCC stage or absence of nodal/distant metastasis as is indicated by the legend. Grey dots indicate samples from normal oesophageal tissue.

First, we asked whether *FOXM1* expression was higher in the OAC-derived samples, and we found it to be expressed to significantly higher levels across cancer samples (Additional file 2: Figure S2A; Figure 3). This is in agreement with our previous observations in a different collection of OAC biopsies [8]. Next, the expression of the FOXM1 target gene cohort was clustered according to expression similarities amongst the genes themselves and also amongst patient samples (Figure 3; bottom panel). The expression values of MMB/DREAM complex components were then superimposed on top of the resulting heatmap and clustered according to similarity of expression only (Figure 3; top panel). Clustering analysis provided a good separation of normal and tumour samples according to their gene expression profiles with only three OAC cases clustering with the normal samples and conversely one normal sample with the OAC samples. Two separate clusters were observed for tumour samples. However, no co-clustering of clinical features was observed amongst the OAC samples (Figure 3, top rows). Nevertheless, the FOXM1 target gene network was clearly a good predictor of the presence of OAC. To determine the generality of these findings, we also analysed the expression of the same set of FOXM1 target genes and MMB/DREAM complex components in a microarray study across a different cohort of patients [4]. Again, the FOXM1 target gene cohort was clustered according to expression similarities amongst the genes themselves and also amongst patient samples (Additional file 2: Figure S2; bottom panel) and a good separation between normal and tumour samples was obtained. Thus, the FOXM1 target gene expression profile provides a good overall indicator of OAC presence but is not diagnostic of any particular clinical feature of disease severity.

**Figure 4:**
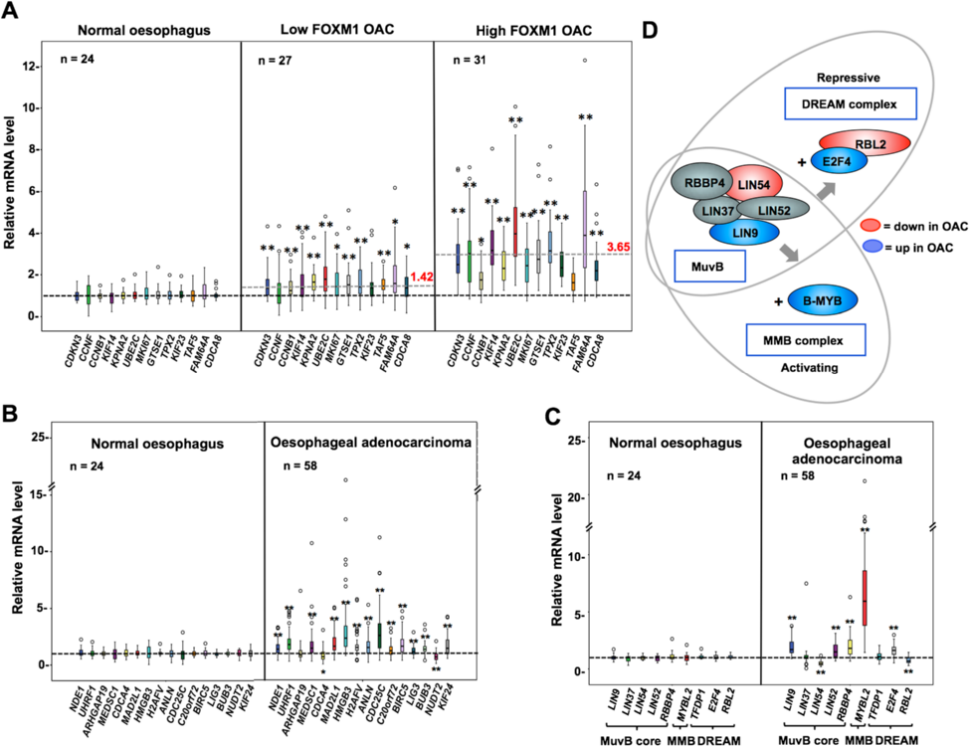
**Box plot representation of the expression of FOXM1 regulatory network genes in OAC patient samples. (A-C)** Box plots of mRNA levels of the FOXM1 target genes in normal oesophageal (left panels) and oesophageal adenocarcinoma (OAC) tissue samples (right and middle panels). Where indicated, the OAC samples are further partitioned according to high (right panel; n = 31) or low (middle panel; n = 27) *FOXM1* expression. High *FOXM1* expression was defined as mRNA levels that were greater than two standard deviations and greater than two-fold of the mean *FOXM1* level in the normal tissues. Genes are grouped according to representing *FOXM1* targets shared between OE33 and U2OS cells (A), specifically bound in OE33 cells (B) or encoding components of the MMB and DREAM complexes (C). The mRNA level relative to the median level of the normal tissues (taken as 1) is shown. Boxes represent the interquartile range and the median value is indicated by the horizontal line. Open black circles represent outliers. The dotted line in (A) is the average median expression value of all the genes in the particular sub-panel (value indicated in red). Statistical significance of the change in expression between OAC and normal tissue, normal and low *FOXM1* OAC tissue or low *FOXM1* and high *FOXM1* OAC tissue, is indicated in the rightmost panel of the two panels being compared (** P-value <0.01; * P-value <0.05). **(D)** Summary of the changes in expression of genes encoding MMB/DREAM complex components in OAC samples (red and blue represent down and up regulated in OAC respectively).

Next, correlations amongst FOXM1 targets with FOXM1 expression were examined and three broad clusters were identified (Figure 3; vertical clustering). Cluster A contains genes that show strong co-association with FOXM1 expression in OAC and show clear evidence of upregulation compared to normal samples. Interestingly, this cluster contains all but one of the genes that we tested which show binding of FOXM1 across different cell types (Figure 3; orange dots. eg *CCNB1* and *UBE2C*) and this pattern is also seen in the independent microarray data set (Additional file 2: Figure S2). Indeed further testing of genes commonly occupied across cell types shows that the majority of these are significantly upregulated across OAC samples (Additional file 2: Figure S3B). Moreover, there was a clear stepwise relationship between the magnitude of *FOXM1* overexpression and the expression levels of members of this group of target genes in OAC (Figure 4A) suggesting a causal link.

The genes in cluster B also show evidence of upregulation in OAC, although the changes compared to normal samples are less marked (Figures 3 and 4B). Again, there is a clear relationship between the levels of *FOXM1* expression and the magnitude of expression of genes within this subset exemplified by *HMGB3* and *CDC25C* (Additional file 2: Figure S4A).

Finally cluster C contains two sub-categories of genes (Figure 3), one of which shows little differences between normal and tumour samples. The second subcategory contains a reciprocal relationship between normal oesophagous and OAC where expression is reduced in the tumour samples, and hence shows an anti-correlation with *FOXM1* expression, despite representing direct targets (eg *AGFG2* and *CCDC85C*)(Figure 3 and Additional file 2: Figure S4B). Collectively, these data show that FOXM1 target gene expression is generally upregulated alongside *FOXM1* expression, although different subclusters can be identified with distinct expression profiles. Although we see this association with *FOXM1* expression, the signatures that we observe might be reflective of the fact that the cells within OACs are cycling more rather than FOXM1 being a driving factor. We therefore also analysed a group of genes previously shown to be regulated at the G1-S transition [21]. Modest increases in expression across this cohort in OAC were seen with the expression of the key marker of the G1-S transition *CCNE1* barely altered in cancer samples and *SERPINB3* being significantly downregulated (Additional file 2: Figure S3C), indicating that there are not necessarily more cells in the cell cycle in OAC tissues.

Having established that FOXM1 and its target genes show strong co-association, we next turned to its coregulators in the MMB complex and the antagonistically acting components of the DREAM complex. We predicted that either there should be no change in their expression or there should be concomitant changes with *FOXM1* expression ie activating components should go up and/or repressive components should go down in cancer samples. Unexpectedly we saw components of the core MuvB complex both increase (eg *LIN9*) and decrease (eg *LIN54*) in expression in OAC samples (Figures 3 and 4C). Similarly, we also saw DREAM-specific components both increase (eg *E2F4*) and to some extent, decrease (eg *RBL2*) across cancers (Figures 3 and 4C). The MMB-specific component *MYBL2* was significantly upregulated across OAC samples (Figures 3 and 4C), and the degree of overexpression generally followed the overexpression levels of *FOXM1* (Additional file 2: Figure S4C). A similar correlative pattern was seen for *LIN9* and *E2F4* whereas the expression of *LIN54* was weakly anti-correlated with *FOXM1* expression levels (Additional file 2: Figure S4C). Importantly we found the same correlative changes in MMB/DREAM complex component expression in the microarray analysis of an independent set of OAC samples (Additional file 2: Figure S3D), although the overexpression of *MYBL2* in these tumours was less marked. Given these close associations between FOXM1 expression and members of the MMB and DREAM complexes, we also determined whether the expression of one of the novel FOXM1 target genes that we identified, *UHRF1*, also correlated with changes in expression of components of these complexes. Positive correlations were seen with *FOXM1*, *MYBL2*, *LIN9* and *E2F4* which all increase in OAC, whereas a weaker negative correlation was seen with *LIN54* (Additional file 2: Figure S4D). Collectively, these findings demonstrate some anticipated outcomes eg co-overexpression of activating components of the MMB complex but also some unexpected discoveries such as upregulation of DREAM-specific or downregulation of MuvB core complex components (summarised in Figure 4D).

### FOXM1 directly binds to LIN9

The co-upregulation of *LIN9* and *MYBL2* with *FOXM1* in OAC patient samples suggested that one or both of the proteins encoded by these genes might functionally interact with FOXM1 either in the context of the entire MMB complex or in subcomplexes. In the latter case, direct interactions with FOXM1 would be anticipated. Previous results have demonstrated that FOXM1 binds to the MMB complex and this results in FOXM1 recruitment to chromatin [16,17]. However, it was not clear which subunit(s) is responsible for binding to FOXM1 and thereby nucleating its recruitment.

**Figure 5:**
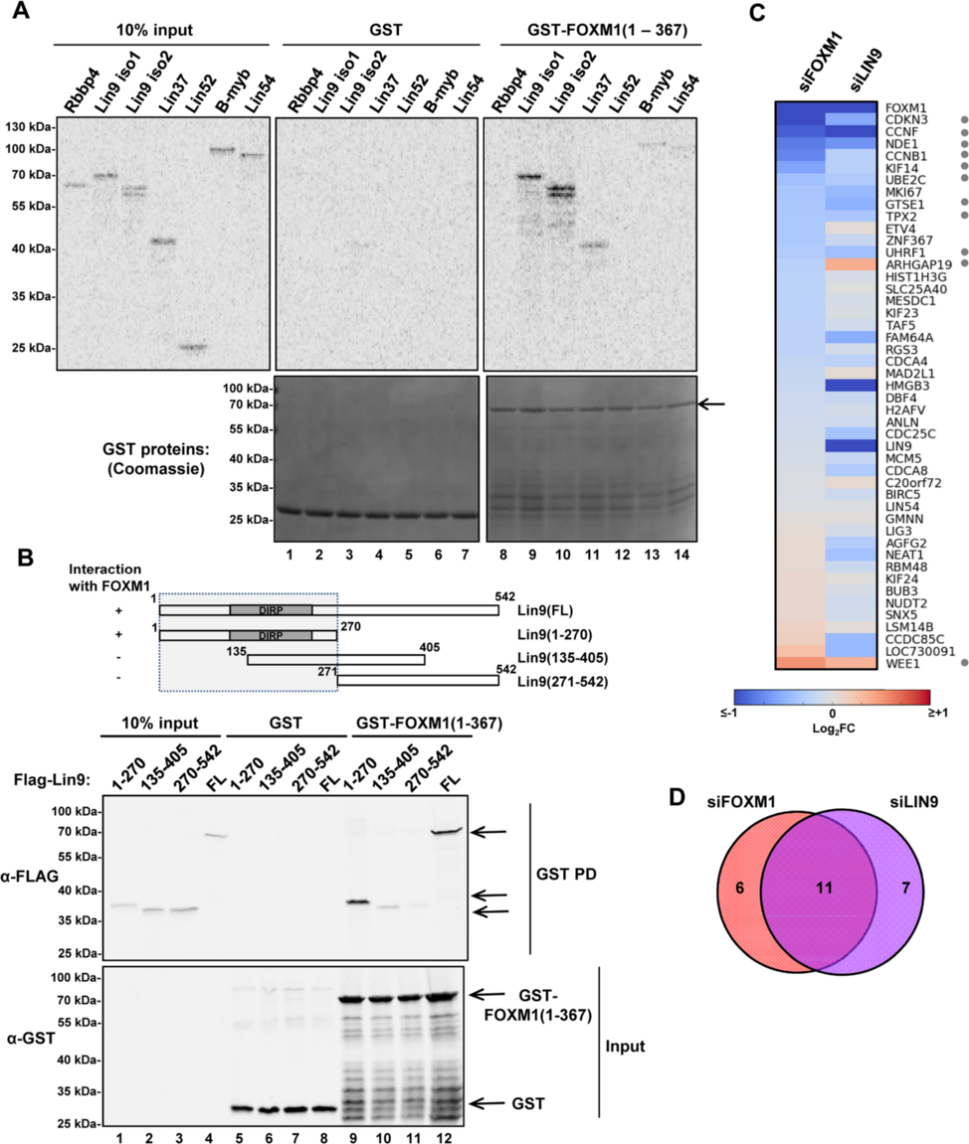
**FOXM1 and interactions with the MMB complex. (A)** GST pulldown analysis using GST or GST-FOXM1(1–367) and the indicated *in vitro* translated MMB and DREAM complex components. Precipitated and input *in vitro* translated proteins were detected by phosphorimaging (top panel) and GST fusion proteins were detected by Coomassie blue staining (bottom panels). Arrow represents the position of the band corresponding to full-length GST-FOXM1(1–367). **(B)** GST pulldown analysis using GST or GST-FOXM1(1–367) and the indicated bacterially expressed and purified full-length and deleted Flag-tagged Lin9 proteins (shown diagrammatically at the top). FOXM1 and Lin9 derivatives were detected by immunoblotting with anti-Flag (top) or anti-GST antibodies (bottom). The region of Lin9 which is sufficient for FOXM1 binding is boxed. **(C)** Nanostring nCounter gene expression assay of the indicated control or FOXM1 target genes following siRNA-mediated knockdown of *FOXM1* or *LIN9*. Data are shown as a heat map of fold change (log_2_) relative to a non-targeting siRNA control and are the average of three independent experiments. Grey dots represent the identities of genes in the overlap of the Venn diagram in part (D). **(D)** Venn diagram of genes showing significant (p < 0.05, t-test) changes in expression following knockdown of one of the two indicated factors. Note that *ARHGAP19* changes in opposite directions for each knockdown.

To determine whether either LIN9 or MYBL2/B-myb interacted with FOXM1, we tested FOXM1 binding to *in vitro* translated individual MMB complex components using a GST pulldown assay with GST-FOXM1(1–367). This N-terminal region of FOXM1 was previously shown to be sufficient for binding to the MMB complex [16]. Strong binding was only consistently obtained to two different isoforms of Lin9 with weak or no binding observed to MYBL2/B-myb or other MMB components (Figure 5A, lanes 9 and 10). To determine whether the interaction is direct, we repeated the assay with Lin9 expressed and purified from bacteria. Strong binding was observed between FOXM1 and Lin9 (Figure 5B lane 12). Further mapping experiments demonstrated that FOXM1 interacts with the N-terminal region of Lin9 encompassing the DIRP (Domain in Rb-related Pathway) domain (Figure 5B, lane 9).

Having established LIN9 as the direct binding partner of FOXM1, we depleted LIN9 in OE33 cells and tested the expression of a subset of FOXM1 target genes. Generally, LIN9 depletion resulted in downregulation of FOXM1 target genes with many being commonly downregulated upon FOXM1 depletion (Figure 5C and D). However, there were differences in the magnitudes of decrease observed in individual cases with genes like *HMGB3* being more sensitive to *LIN9* depletion and *CDKN3* being more sensitive to *FOXM1* depletion (Figure 5C).

Collectively, these data show that FOXM1 and LIN9 interact directly and co-regulate a similar set of genes. This helps provide a molecular rationale for why we observe co-upregulation of the genes encoding these two transcriptional regulators in OAC patients.

### Clinical insights from the FOXM1 regulatory network

**Figure 6:**
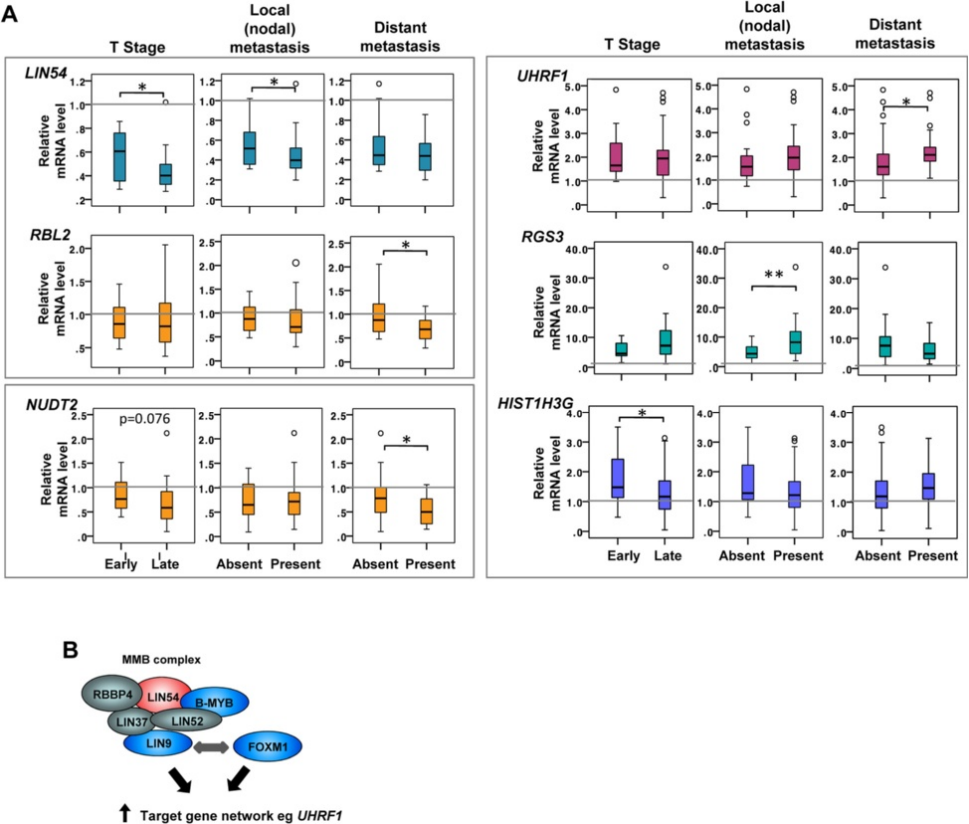
**Changes in the FOXM1 target gene network in late stage OAC. (A)** Box plots of mRNA levels of the indicated genes in OAC tissue samples grouped according to T stage, the presence of local metastases (absent, n = 18; present, n = 37) or the presence of distal metastases (absent, n = 38; present, n = 18). Early and late T stage was defined as stage 1 or 2 disease (n = 16) and stage 3 or 4 disease (n = 32) respectively. The mRNA level relative to the median level of normal tissues (taken as 1; grey lines) is shown. Boxes represent the interquartile range and the median value is indicated by the horizontal line. Open black circles represent outliers. Statistical significance is indicated (** P-value <0.01; * P-value <0.05). **(B)** Summary of the changes in expression of *FOXM1* and genes encoding MMB/DREAM complex components in OAC samples (red and blue represent down and up regulated in OAC respectively) leading to general upregulation of the FOXM1 target gene network.

It is clear that the expression of *FOXM1*, several of its co-regulatory partners and many of its target genes are of potential diagnostic use for identifying the presence of OAC. To further interrogate our data, we subdivided our OAC samples as either early or late T stage, and also whether metastases (local or distant) were present in the patients. First we analysed the expression of MMB and DREAM complex components and found that amongst tumours, reduced levels of *LIN54* are generally found in tumours (Figure 4C) but lower levels are indicative of late stage disease in patients with late T stage tumours and local metastases (Figures 4C and 6A). A similar trend was seen for *RBL2*, except that in this case, reduced expression was significant in patients with distant metastases (Figure 6A). Other components of these complexes which showed upregulation in OAC samples, including *FOXM1* itself, did not differ in their expression between patients with early or late stage cancer (Additional file 2: Figure S5A). The lack of stage-specific changes in *FOXM1* expression is consistent with our previous data [8]. Thus while the expression of many MMB and DREAM complex components is not changed according to disease stage, reduced expression of a core component of the MuvB complex (*LIN54*) and a DREAM-specific component (*RBL2*) are good indicators of the presence of late stage disease.

Next, we examined the expression of members of the FOXM1 target gene network across patients from different disease stages. In general the expression of FOXM1 target genes did not associate with disease severity. Indeed, we were unable to find significant stage-specific differences in expression in any of the FOXM1 targets that were shared between OE33 and U2OS cells (data not shown). However, several genes in the “OE33-specific” FOXM1 target gene exhibited differential expression according to disease stage. For example, *UHRF1* and *RGS3* are both overexpressed in general in cancers (Figure 4B; Additional file 2: Figure S4B) but they showed evidence of increased expression in late stage disease (significantly enhanced in patients with distant and local metastases respectively) (Figure 6A). *UHRF1* is particularly interesting in this context given its recent identification as an oncogene that drives DNA hypomethylation in cancer cells [22]. In contrast, *HIST1H3G* showed significantly reduced expression in patients with late T stage disease (Figure 6A) and although generally highly expressed in OAC (Figure 4B; Additional file 2: Figure S4B), both *C20orf72/MGME2* and *SNX5* showed significantly reduced expression when local metastases are present (Additional file 2: Figure S5B). Interestingly, we also found that *NUDT2* whose expression is generally decreased in OAC (Figure 4B) shows reduced expression in late stage disease, which reaches statistical significance in patients with distant metastases (Figure 6A). Together these results identify several FOXM1 target genes whose expression changes in OAC, which are of potential value in predicting the presence of late stage disease.

## Discussion

FOXM1 overexpression has been observed in a variety of different tumour types (reviewed in [7]) and our recent work indicated that in the context of OAC, several of its target genes, including *PLK1*, are co-ordinately overexpressed in this cancer type [8]. However, it was not known how FOXM1 contributes to tumourigenesis in this context. To address this issue, we have extended these studies to a broader cohort of FOXM1 target genes, the majority of which are novel targets and have not previously been studied in the context of cancer. Here we demonstrate that there is a more widespread co-ordinate upregulation across the FOXM1 target gene network in OAC.

FOXM1 has previously been implicated in cell cycle control [10] and many of its target genes have known or suspected functions in the late G2 and early M phase [16,17,19]. Many of this class of target genes are efficiently bound by FOXM1 in both OE33 cells and the non-OAC U2OS cells (eg *CCNB1* and *CENPF*), indicative of a core function for FOXM1 across cell types. This class of target genes is generally co-upregulated with FOXM1 in OAC. However, there are another set of FOXM1 target genes, typified by *HIST1H3G*, that are more efficiently bound by FOXM1 in OE33 cells, suggesting a more cell type-specific activity for FOXM1. Many of these genes are also co-ordinately deregulated with FOXM1 in OAC, and in the case of *HIST1H3G*, a lowering of expression towards the levels found in normal tissue is indicative of late stage disease. Reciprocally, other targets like *RGS3* are generally upregulated in OAC but show higher levels in tumours with local metastases. Thus, FOXM1 has potentially acquired new tissue/cell type specific functions in OAC in keeping with the novel recently identified roles of FOXM1 in gliomas, breast cancer and lymphoblastomas through targeting different gene networks in each tumour type [13-15]. In the latter cases, FOXM1 interacts with different transcription factors to elicit its novel effects but it is unclear whether re-direction of FOXM1 targeting is driven by any particular transcription factor in OAC or whether other mechanisms might be operative.

The majority of the FOXM1 target genes that we have discovered and investigated in the context of OAC are novel target genes and have not been extensively studied in the context of cancer. One such gene that stands out is *UHRF1* which has been shown to act as an oncogene that drives DNA hypomethylation in hepatocellular carcinoma [22]. *UHRF1* encodes a multi-domain protein involved in histone ubiquitination and recruiting the DNA methylase DNMT1 to chromatin during DNA replication, and hence plays a pivotal role in sculpting the epigenetic landscape [23]. *UHRF1* had not previously been linked to FOXM1 or OAC but has been shown to be overexpressed in a wide range of other cancers [23] and consistent with our observations of high levels in late stage OAC, has also been shown to be a marker for tumour aggressiveness in cervical cancer [24]. Among the other genes that we linked to late stage disease, little is known about their potential roles in other cancers although *NUDT2* has previously been shown to be upregulated in human breast carcinomas [25] whereas *SNX5* is upregulated in papillary thyroid carcinomas [26]. Mechanistically, we have demonstrated that in addition to FOXM1 binding to their regulatory regions, FOXM1 and LIN9 are important activators of many of these target genes (Figures 2B and 5C & D) indicating that the LIN9-FOXM1 complex plays an important role in upregulating these genes in OAC.

In addition to studying the FOXM1 target gene network, we also studied the expression of MMB complex components in OAC. Previous studies have implicated FOXM1 and MYBL2 overexpression in a range of different cancers (reviewed in [18]). In contrast little is known about the expression of other core complex components in cancers although LIN9 is part of the Mammaprint breast cancer profile which is prognostic for metastatic disease [27]. The MMB complex works synergistically with FOXM1 to drive cell cycle gene expression [16,17], it was expected that either there would be no change or alternatively co-ordinate upregulation of the MMB complex components. In the first scenario, FOXM1 could use pre-existing MMB complex components to target the same genes more efficiently and/or excess FOXM1 might then participate in new interactions and hence deregulate a new target gene network. Overexpression of the entire MMB complex would presumably facilitate FOXM1 recruitment to cell cycle genes. However, although we found co-ordinate upregulation of the MMB-specific component *MYBL2* with *FOXM1*, the MuvB core subunit components *LIN9* and *LIN54* were differentially expressed with *LIN9* being co-ordinately upregulated and LIN54 being downregulated in OAC (Figure 6B). Ultimately, this series of events coincides with the upregulation of the FOXM1 target gene network and suggests a co-operative mode of action. These findings on a central FOXM1-LIN9-MYBL2/B-myb axis are consistent with the recent finding that the human papillomavirus E7 protein controls mitotic gene activation through interacting with FOXM1, MYBL2/B-Myb and LIN9 components of the MMB complex [28]. However while *LIN9* and *MYBL2* upregulation in OAC might be explicable, the downregulation of *LIN54* is entirely unexpected. Both observations suggest that in the context of OAC, different MMB-like complexes might be forming with different stoichiometries which likely contribute to the differential gene expression programmes we observe. Importantly, we show that amongst MMB complex components, LIN9 is the one that is directly bound by FOXM1. Thus, there is the potential for the assembly of complexes containing these two components in OAC. It is currently unclear which other components are recruited in the context of OAC, but presumably the reductions in *LIN54* levels suggest it is unlikely that LIN54 will play a part in driving OAC. In OAC-derived cells, both FOXM1 and LIN9 co-ordinately activate a subset of FOXM1 targets, consistent with the observation that these interact directly and are co-overexpressed in OAC, further supporting a co-regulatory role in the context of OAC. However the role of LIN54 remains enigmatic as it is also required for efficient FOXM1 target gene expression in OE33 cells (data not shown) and yet shows an anti-correlation with FOXM1 target gene expression in OAC samples. It is possible that the overexpression of LIN9 and/or MYBL2 might over-ride the requirement for LIN54 in this context. LIN54 has previously been implicated in targeting the MMB complex to DNA [29], thus it is possible that the loss of LIN54 might allow binding of FOXM1-MMB subcomplexes to alternative regulatory regions, either directly or through different DNA binding proteins.

Collectively our data show that both the FOXM1 target gene network and its co-regulatory partner proteins are deregulated in OAC. The expression of the FOXM1 target gene network is strongly predictive for the presence of OAC. Similarly the expression of FOXM1 itself and several of its co-regulatory partners show good predictive power for diagnosing OAC. While the networks do not give prognostic power, the expression of several of the target genes we have studied provide indications of disease stage.

## Materials and methods

### Tissue collection and cell lines

Ethical approval for collection of oesophageal tissue samples from patients at the Royal Albert Edward Infirmary, Wigan and the Salford Royal Hospital were granted by the ethics committees at Wrightington, Wigan and Leigh NHS Foundation Trust (2007) and Salford Royal NHS Foundation Trust (2010) respectively.

Biopsy tissue samples (~4 mm) were preserved in RNAlater (Qiagen) or immediately snap-frozen in liquid nitrogen and archived at −80°C. Normal control samples were collected from patients with no macroscopic evidence of oesophageal cancer. Patient demographics and clinical details were collected. Patients were staged according to standard specialist multidisciplinary team (MDT) practice with computed tomography (CT), endoscopic ultrasound (EUS) and positron emission tomography (PET) as appropriate. Tumour stage was classified according to the updated 7th Edition of the American Joint Committee on Cancer (AJCC) staging system [30].

OE33 and U2OS cell lines were grown as described previously [16,31].

### RNA isolation and Nanostring nCounter expression analysis

Total cellular RNA was isolated from cell line and clinical tissue samples as described previously [31]. When required, short interfering (si) RNAs directed against human *FOXM1*, *LIN9* and *LIN54* (SMARTpools; Dharmacon), or a non-targeting pool (Dharmacon) were used. Cells were transfected using Lipofectamine RNAiMAX transfection reagent (Invitrogen) and siRNA treatment was performed with 100 pmol for 24 hrs prior to mRNA expression analysis. Sample hybridization, purification, immobilisation and imaging were performed according to the manufacturer’s protocol (Nanostring Technologies).

Digital Analyser output reporter code count (RCC) files were analysed using the nSolver Analysis software (Nanostring Technologies), using default settings. Inbuilt data analysis workflow wizards were used to perform quality control, positive control normalisation and reference gene normalisation on the data. For each analysis mRNA counts were normalised by positive control spike-in probes supplied with the CodeSet and by the geometric mean of the internal reference genes. Cell line data was normalised using *ALAS1*, *GAPDH* and *HMBS* internal reference genes. Five reference genes (*SDHA*, *ALAS1*, *GAPDH*, *HMBS* and *PARPBP*) were used to normalise the clinical samples data.

In knockdown experiments statistical significance was calculated using an unpaired two-tailed Student’s T test with a two sample equal variance. Gene expression data comparing expression in different groups of clinical tissue samples were represented with boxplots generated using SPSS Statistics v20 software (IBM). Outliers represent values >1.5 interquartile ranges from the 25^th^ or the 75^th^ percentile (e.g. 75^th^ percentile + (1.5 × IQR) and 25^th^ percentile – (1.5× IQR)). Statistical significance was assessed by the Mann Whitney *U* Test calculated using SPSS Statistics v20. Heatmaps of gene expression in clinical tissues quantified using the Nanostring nCounter system were produced using the pheatmap: Pretty Heatmaps software package in R (http://CRAN.R-project.org/package=pheatmap).

### ChIP and ChIP-seq analysis

ChIP-qPCR and ChIP-seq were carried out as described previously [16]. For ChIP-seq, 3×10^7^ cells, 3 μg antibody (FOXM1; Santa Cruz Biotechnology (sc- 502 X) or rabbit IgG; Millipore (12–370)) and 30 μl Dynabeads were used per experiment. Library preparation was performed using the TruSeq ChIP Sample Preparation Protocol (Illumina) and DNA libraries were sequenced using the Genome Analyser IIx (Illumina).

### Plasmids, protein purification and GST pulldown assays

pAS3091 (pET-30b-Lin9(1–270)), pAS3092 (pET-30b-Lin9(135–405)), pAS3093 (pET-30b-Lin9(271–542)) or pAS3094 (pET-30b-Lin9(full-length)) were created by inserting Nde1/XhoI-cleaved PCR products (created using the primer pairs ADS3727/ADS3731, ADS3728/ADS3732, ADS3729/ADS3730 and ADS3727/ADS3730 respectively, and pAS3078 as a template) into the same sites in pET-30b.

GST-tagged FOXM1(1–367) protein was purified as described previously [16]. To purify His tagged Lin9 proteins, BL21-CodonPlus-RIL bacteria were transformed with the following plasmids: pAS3091 (encoding Lin9(1–270)), pAS3092 (encoding Lin9(135–405)), pAS3093 (encoding Lin9(271–542)) or pAS3094 (encoding full-length Lin9). His-tagged proteins were purified using Ni-Agarose (Qiagen) essentially according to the manufacturer’s instructions followed by dialysis into 1× PBS. Glycerol was added to 30% final concentration for storage at −80°C.

To make *in vitro* translated proteins, the following plasmids were used (kindly provided by Kurt Engeland): pAS3077 (pcDNA3.1-RbAp48), pAS3078 (pcDNA3.1-Lin9 isoform 1), pAS3079 (pcDNA3.1-Lin9 isoform 2), pAS3080 (pcDNA3.1-Lin54), pAS3081 (pcDNA3.1-Lin52), pAS3082 (pcDNA3.1-Lin37), pAS3083 (pcDNA3.1-E2f4), pAS3084 (pcDNA3.1-B-Myb), and pAS3085 (pcDNA3.1-Dp1). The *in vitro* translation was performed using TNT Quick Coupled Transcription/Translation Systems (Promega) according to the manufacturer’s protocol.

The GST-pull down experiments were performed as previously described [16], using 500 ng of GST-FOXM1(1-367) with 1 μl of IVT proteins or 1/10 of the eluate of the His-tagged Lin9 proteins.

### Bioinformatics analysis

Sequencing tags/reads from the FOXM1 ChIPseq experiment in OE33 cells were aligned to the NBCI Build hg18 of the human genome with Bowtie v0.12.7 [32]. Up to two mismatches were allowed. Only reads that uniquely mapped to the genome were preserved. Peak calling was performed with MACS v1.4.2 software [33] using default parameters. To identify high confidence FOXM1 binding peaks, the MACS peak calling output from two experimental replicates was used. Peaks that were identified in both experimental replicates (overlapping peaks) with a false discovery rate (FDR) <10 and a tag density (TD) >15 in at least one of the experimental replicates were identified as significant high confidence peaks. When determining the peak overlaps from each analysis an in-house script was used to determine the percentage of the region of the smaller peak that overlapped with the larger peak. Overlap cut-off threshold was set to 50%, such that 50% of the smaller peak in one replicate was required to overlap with the peak in the other replicate to be considered an overlapping peak. To identify high sensitivity FOXM1 binding events, reads from two experimental replicates were pooled and peak calling was performed again on the combined reads dataset. Peaks with an FDR <10 and TD > 30 were defined as significant high sensitivity peaks. An identical analysis pipeline was performed on the FOXM1 ChIP-seq data from U2OS cells ([16]; GSE38170). Gene annotation was performed using an in-house script to identify the closest gene to the peak summit using the co-ordinates from the Refseq GH 18 v.55 protein coding list. The nearest gene was ascribed to the binding peak when the summit of the peak occurred within 5 kb upstream or 1 kb downstream of the transcription start site (TSS). Additionally if the binding peak was within 1 kb of the promoter region of any gene this was included.

Cis-regulatory element annotation system (CEAS) analysis [34] was performed using the Galaxy/Cistrome web tool (http://cistrome.org/ap/) using the Build 36.3/hg 18 of the human genome and default settings. Gene ontology (GO) analysis was performed using the GREAT web application (http://bejerano.stanford.edu/great/public/html/) [35] using NBCI Build 36.3/hg 18 of the human genome. Tag density heatmaps and profiles were generated using Seqminer v.1.3.3e using default settings (peak extensions 5 kb upstream and 5 kb downstream of the peak summit and bin size 50 bp).

Processing of a microarray dataset profiling gene expression of 28 normal and 64 oesophageal adenocarcinoma samples (accession number: GSE13898) [4] and the calculation of Pearson’s correlation coefficients (PCC) of FOXM1 target genes to FOXM1 expression across tumours was described previously [8]. Clustering and visualisation of the expression levels of the FOXM1 target genes were performed by MultiExperiment Viewer, a part of TM4 microarray software suite [36].

## Additional files

Additional file 1: Table S1. FOXM1 binding regions identified using ChIPseq in OE33 and U2OS cell lines. Data are shown according to classification as high confidence (found in both replicates) or high coverage (found when reads are combined from both replicates and binding regions recalled). Additional file 2:
**Supplementary Figures S1-5.**
Additional file 3: Table S2. Clinical details of the patient samples. Patients are grouped by tissue type (normal oesophageal tissue and oesophageal adenocarcinoma tissue). Basic demographic details are shown for all patients. Median age and interquartile range (IQR) is shown. Clinical staging and treatment details are provided for the oesophageal adenocarcinoma group. The number of cases is shown with percentages in brackets. Individual T, N, M stage and histologic grade of tumour is shown as well as the overall American Joint Committee on Cancer (AJCC) stage using the 2010 staging criteria. The number of cases with missing data is indicated where necessary. One OAC sample was omitted from the final Nanostring analysis as the gene expression changes showed this to be an outlier.
