# Erratum to: ‘Deregulation of the FOXM1 target gene network and its coregulatory partners in oesophageal adenocarcinoma’

**DOI:** 10.1186/s12943-016-0506-6

**Published:** 2016-03-02

**Authors:** Elizabeth F. Wiseman, Xi Chen, Namshik Han, Aaron Webber, Zongling Ji, Andrew D. Sharrocks, Yeng S. Ang

**Affiliations:** 1 Faculty of Life Sciences, University of Manchester, Michael Smith Building, Oxford Road, Manchester, M13 9PT UK; 2 Faculty of Medical and Human Sciences, University of Manchester, Oxford Road, Manchester, UK; 3 Present address: The EMBL-European Bioinformatics Institute, Wellcome Trust Genome Campus, Hinxton, Cambridge, CB10 1SD UK; 4 Present address: Gurdon Institute and Department of Pathology, Tennis Court Road, Cambridge, CB2 1QN UK

After publication of this study [1], we discovered that we had unintentionally omitted to include an accession number for our ChIP-seq data. The ChIP-seq data for FOXM1 in OE33 cells is available at ArrayExpress; accession number E-MTAB-4121. In addition, we omitted to include lists of the high confidence and high coverage FOXM1 binding regions. This has now been amended in a revised Additional file 1: Table S1 which also now features a “legend” tab which more clearly explains the contents of each spreadsheet.

## Additional file

Additional file 1: Table S1. FOXM1 binding regions identified using ChIPseq in OE33 and U2OS cell lines. Data are shown according to classification as high confidence (found in both replicates) or high coverage (found when reads are combined from both replicates and binding regions recalled). (XLSX 519 kb)
