# Supplementary material for: Deregulation of the FOXM1 target gene network and its coregulatory partners in oesophageal adenocarcinoma
Source: Mol Cancer. 2015 Mar 26;14:69. doi: 10.1186/s12943-015-0339-8 (PMC4392876; doi:10.1186/s12943-015-0339-8)
Supplement: Additional file 2: — Supplementary Figures S1-5. [file 12943_2015_339_MOESM2_ESM.pdf]

Supplementary Information

Supplementary figure legends

Supplementary Fig. S1. Validation of FOXM1 ChIP-seq data. (A) Screenshots from the UCSC browser showing the FOXM1 binding peaks for the each of the genes tested in (B). The locations of the PCR products produced in (B) are shown with the green arrows. (B) qPCR quantification of genomic loci corresponding to six peaks associated with OE33-specific FOXM1 target genes. Data are shown for the signal generated by ChIP with a FOXM1 antibody (green bars) compared to non-specific IgG ChIP (purple bars). *FOXO4* negative control region is also shown. The mean percentage of input bound of two independent experiments is shown for each locus. Error bars represent the standard error of the mean. Fold enrichment of each gene locus over *FOXO4* is shown above each column. The tag density of each peak from the combined ChIP-seq experiments is shown below the corresponding column.

Supplementary Fig. S2. Heatmap representation of expression of the FOXM1 regulatory network in OAC patient samples. Heatmap summary of microarray gene expression analysis of direct FOXM1 target genes (bottom panel) or genes encoding members of the MMB and DREAM complex (top panel), in samples from 28 normal and 64 tumour tissues (Kim et al., 2010). The same genes are included as in Fig. 3 which are also present on the microarray. The expression level of each gene is represented by the z-score of the normalised mRNA level across all samples. Blue and cream represent high and low expression respectively as indicated by the scale bar. Rows (representing individual genes) and columns (representing individual tissue samples from normal (ESO) and tumour (EAC)

tissues) are ordered by unsupervised hierarchical clustering of the FOXM1 target genes. Major clusters if genes are indicated (A’-C’) and samples are broadly categorised into clusters of normal or tumour samples. Genes bound by FOXM1 in both OE33 and U2OS cells are marked by an orange dot. The position of FOXM1 is indicated by the black arrow.

Supplementary Fig. S3. Box plot representation of the expression of FOXM1 and its target genes in OAC patient samples. (A-C) Box plots of Nanostring-derived (our patient cohort) mRNA levels of *FOXM1* (A), FOXM1 target genes (B) or a selection of genes expressed at the G1-S cell cycle transition (C) in normal oesophageal (left panels) and oesophageal adenocarcinoma (OAC) tissue samples (right panels). (D) Boxplots of microarray-derived (Kim et al., 2010) mRNA levels of FOXM1 and MMB/DREAM complex components in normal oesophageal (left panels) and oesophageal adenocarcinoma (OAC) tissue samples (right panels). The mRNA levels relative to the median level of the normal tissues (taken as 1) is shown. Boxes represent the interquartile range and the median value is indicated by the horizontal line. Open black circles represent outliers. Statistical significance of the change in expression between OAC and normal tissue is indicated in the rightmost panel of the two panels being compared (** P-value <0.01; * P-value <0.05).

Supplementary Fig. S4. The inter-relationship between the expression of FOXM1, its co-regulators and its target genes in OAC. (A-B) Box plots of mRNA levels of the FOXM1 target genes found specifically in OE33 cells in normal oesophageal (left panels) and oesophageal adenocarcinoma (OAC) tissue samples (right and middle panels). The OAC samples are further partitioned according to high (right panel; n=31) or low (middle panel; n=27) FOXM1 expression. High FOXM1 expression was defined as mRNA levels that were greater than two standard deviations and greater than two-fold of the mean FOXM1 level in

the normal tissues. Genes are grouped according to representing FOXM1 targets whose expression correlates well (r value >7)(A) or relatively poorly (r value <7)(B) with FOXM1 expression in a microarray study of OAC samples (Kim et al., 2010). The mRNA level relative to the median level of the normal tissues (taken as 1) is shown. Boxes represent the interquartile range and the median value is indicated by the horizontal line. Open black circles represent outliers. The dotted line in (A) is the average median expression value of all the genes in the particular sub-panel (value indicated in red). Statistical significance of the change in expression between OAC and normal tissue is indicated in the rightmost panel of the two panels being compared (** P-value <0.01; * P-value <0.05). (C-D) Scatterplots of *FOXM1* (C) or *UHRF1* (D) expression in OAC samples versus the expression of the indicated MMB or DREAM complex components. R^2 values for correlations in expression are shown.

Supplementary Fig. S5. Changes in the FOXM1 target gene network in late stage OAC. Box plots of mRNA levels of MMB complex components (A) or the indicated FOXM1 target genes (B) in OAC tissue samples grouped according to T stage, the presence of local metastases (absent, n=18; present, n=37) or the presence of distal metastases (absent, n=38; present, n=18). Early and late T stage was defined as stage 1 or 2 disease (n=16) and stage 3 or 4 disease (n=32) respectively. The mRNA level relative to the median level of normal tissues (taken as 1; grey lines) is shown. Boxes represent the interquartile range and the median value is indicated by the horizontal line. Open black circles represent outliers. Statistical significance is indicated (* P-value <0.05).

A

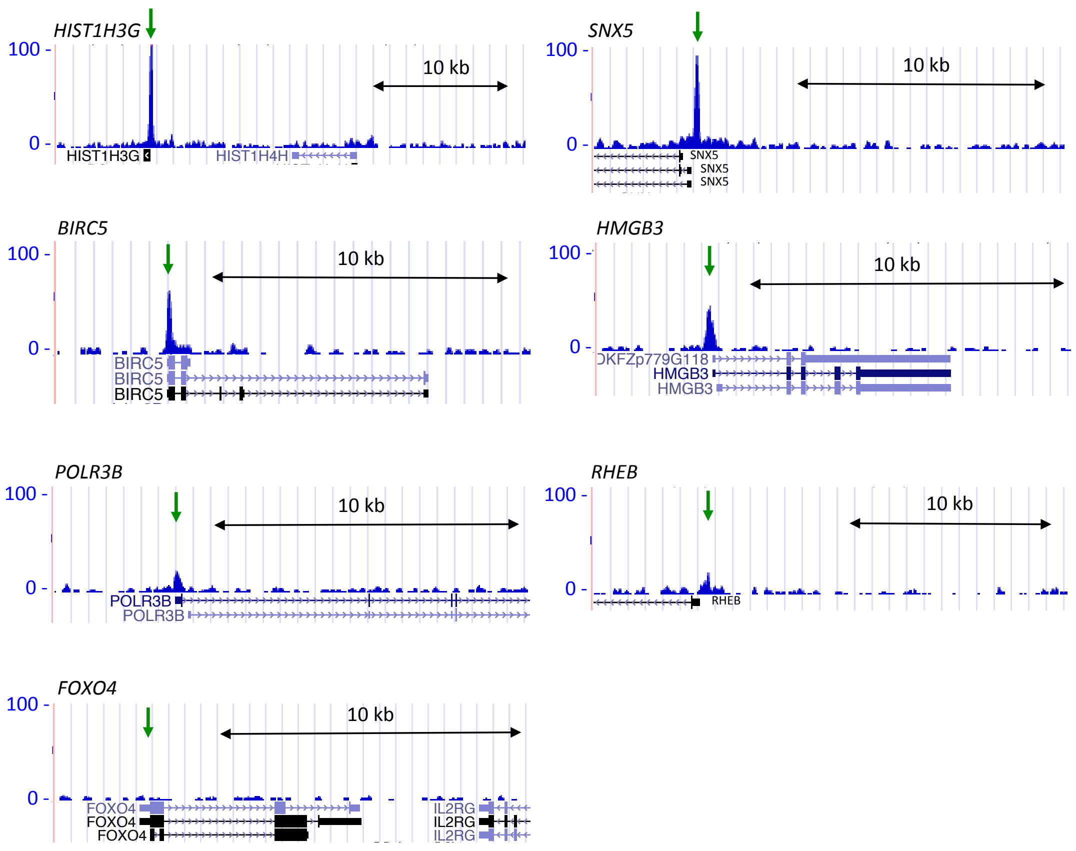

B

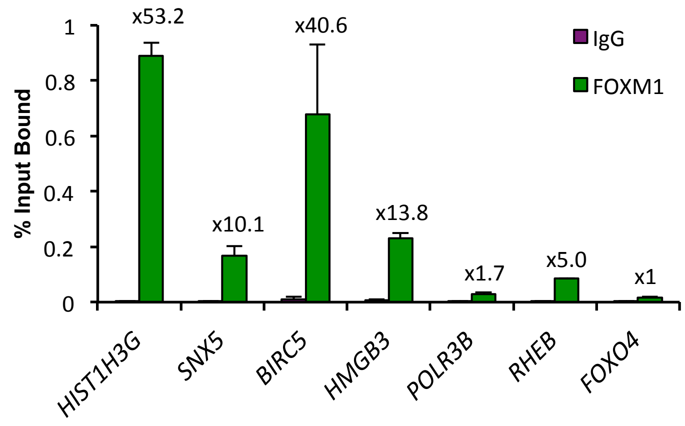

Tag Density	137	95	48	52	25	25	N/A

Supplementary Fig. S1 Wiseman et al., 2014

A

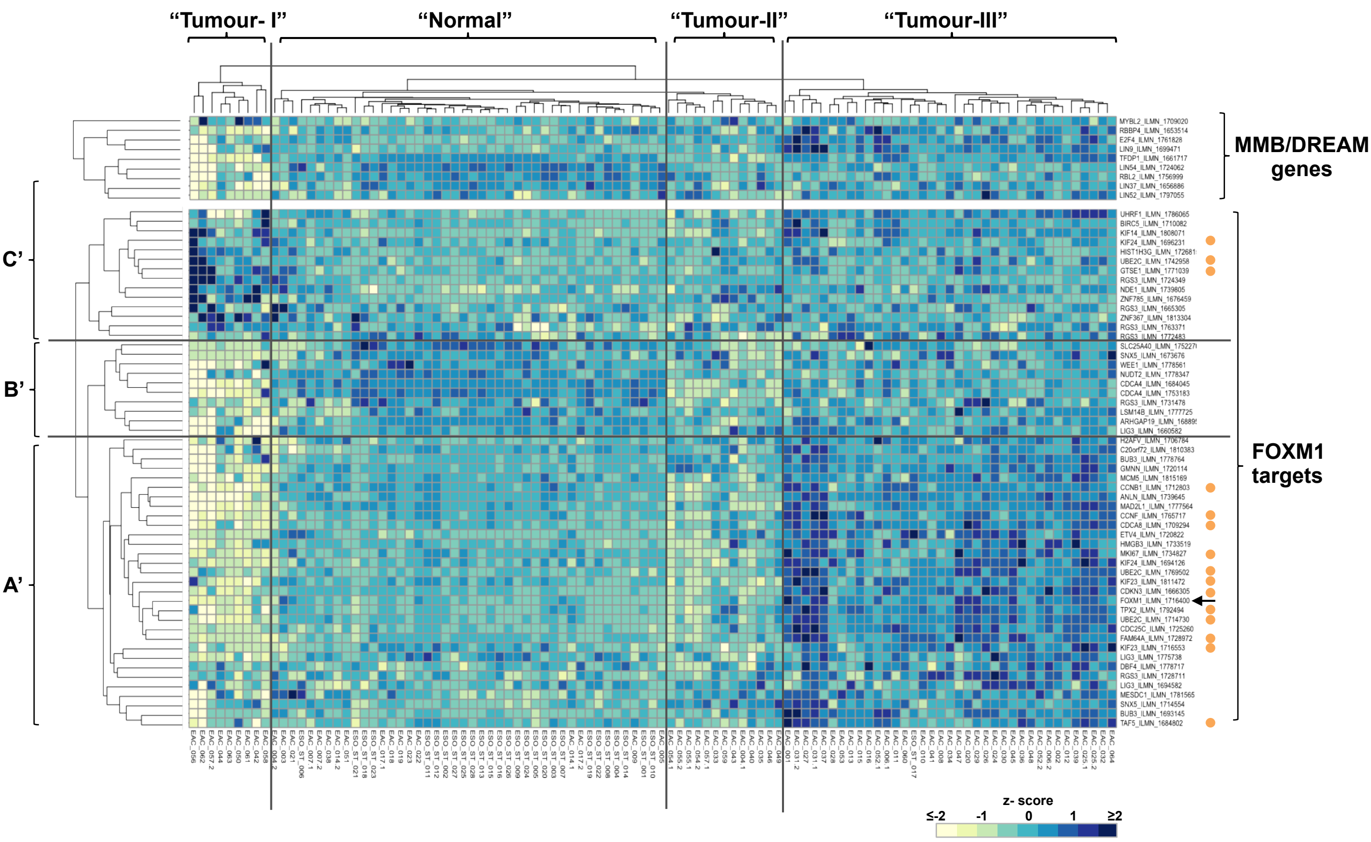

Supplementary Fig. S2 Wiseman et al., 2014

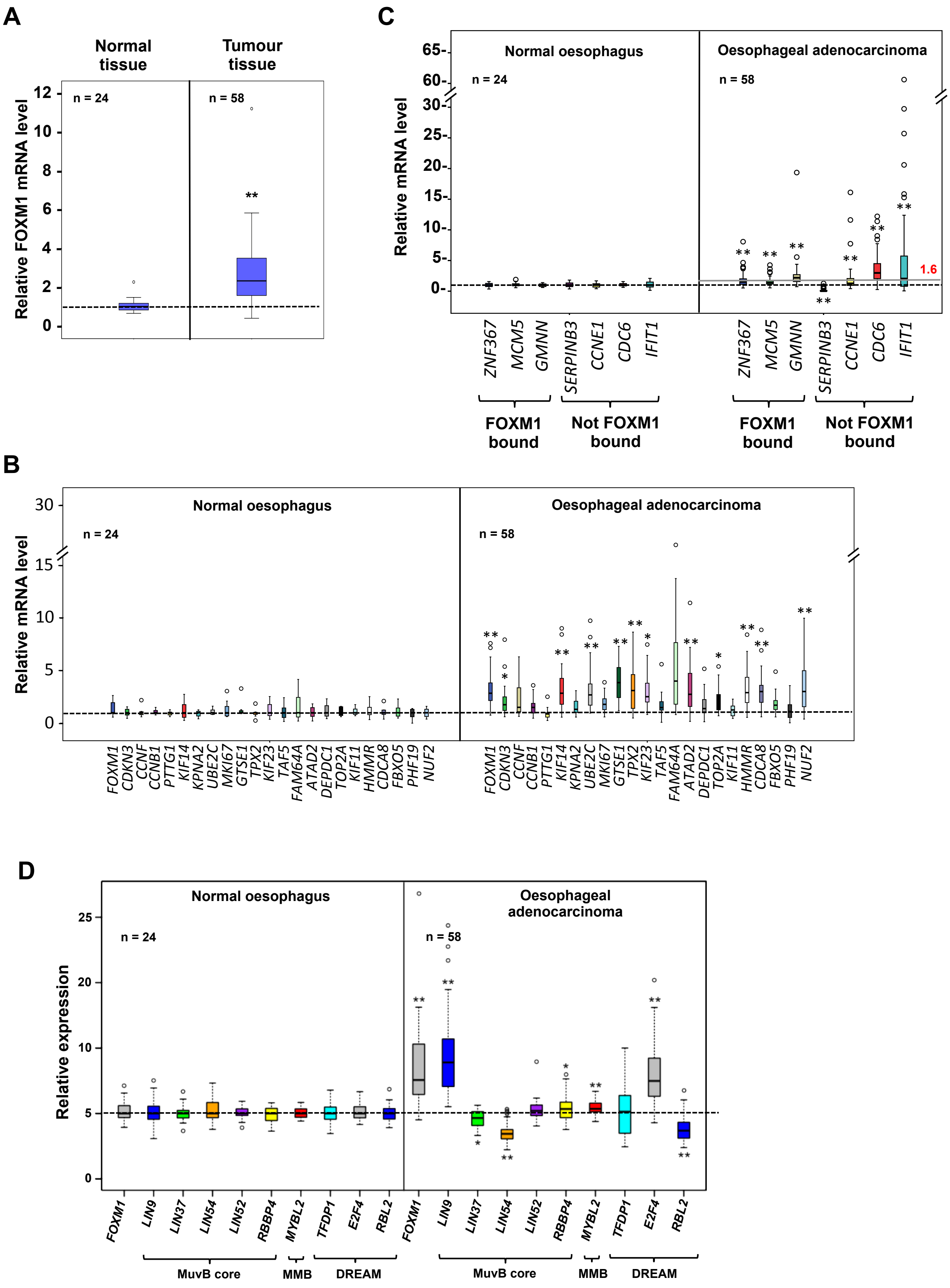

Supplementary Fig. S3 Wiseman et al., 2014

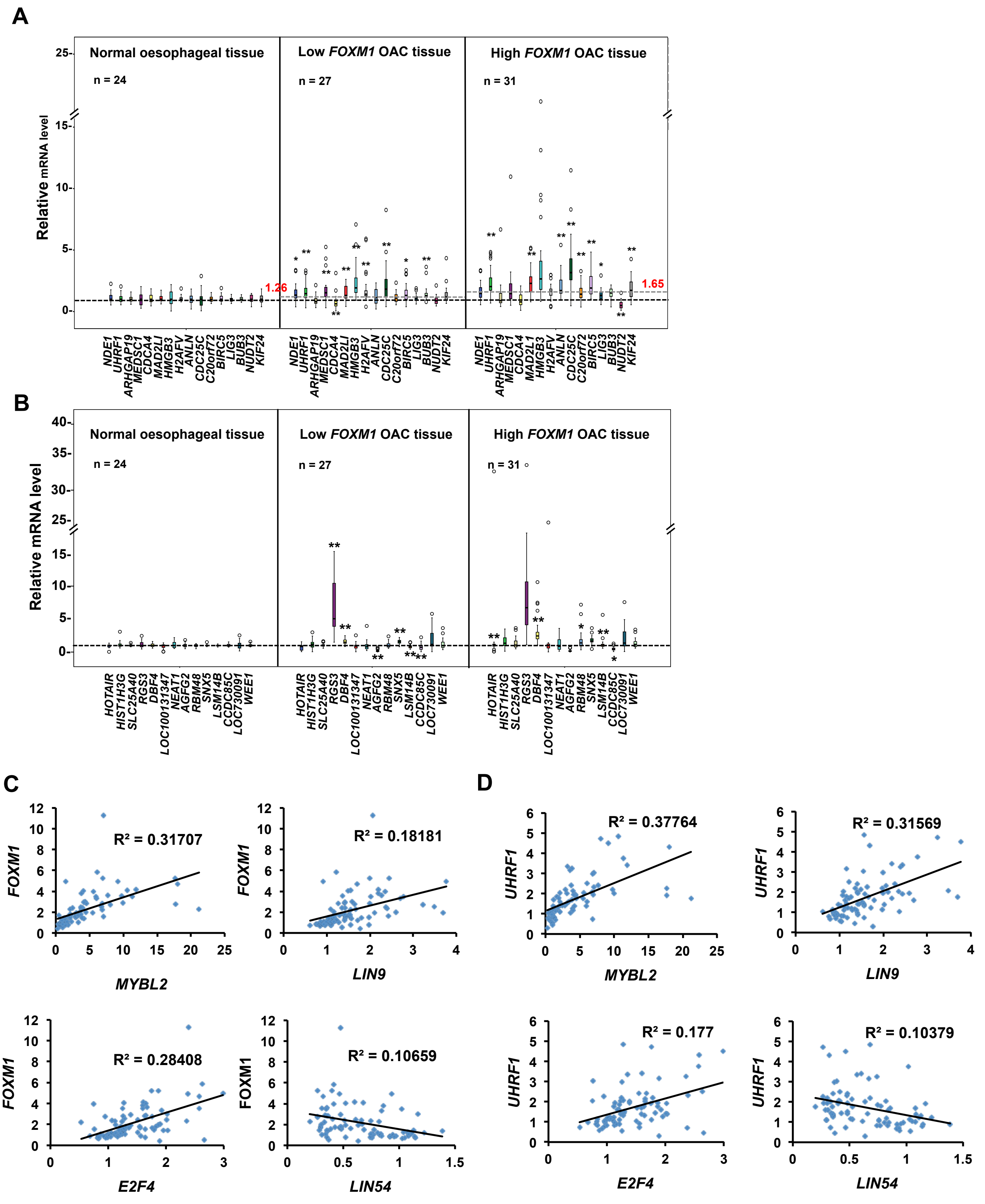

Supplementary Fig. S4 Wiseman et al., 2014

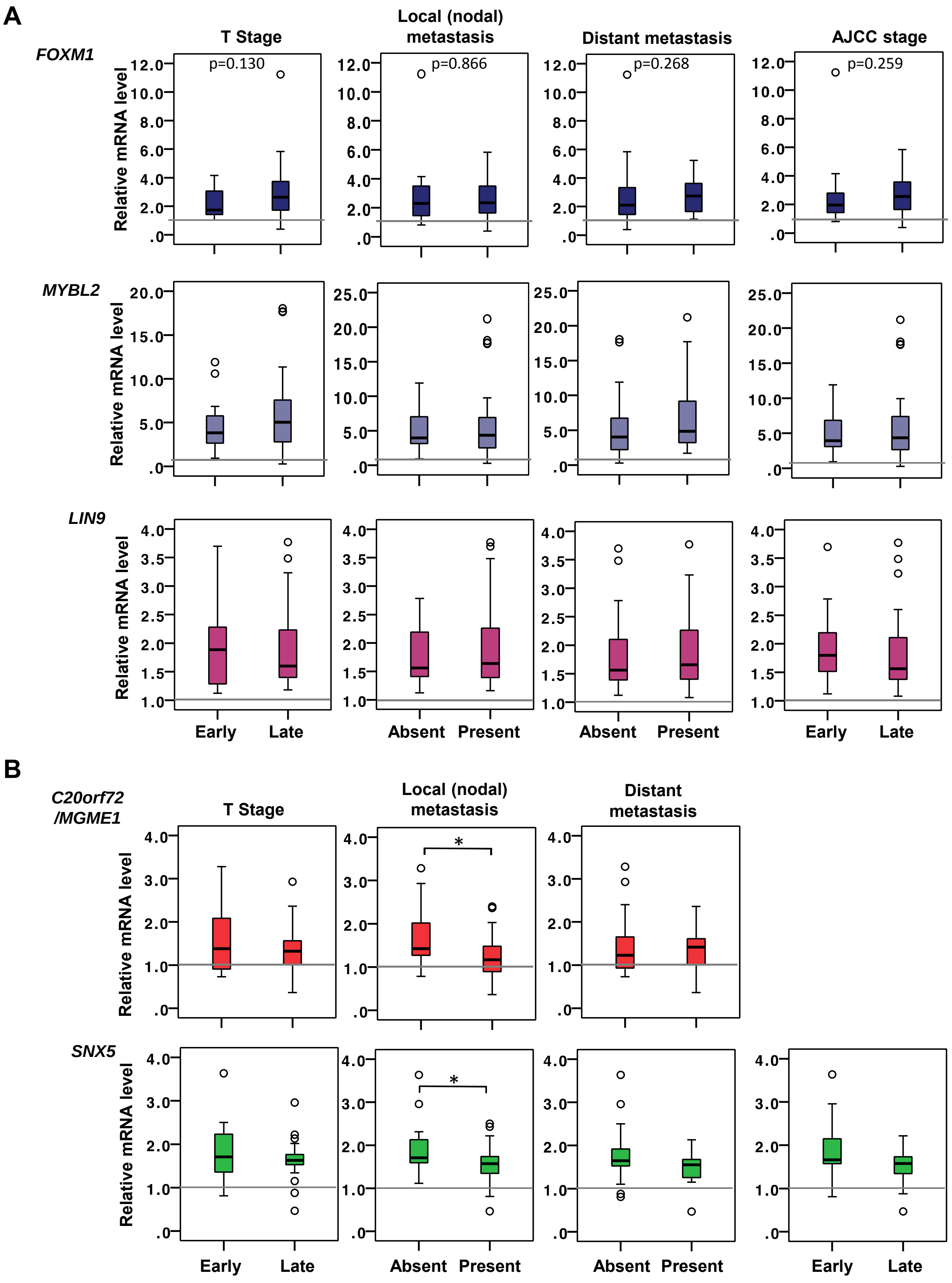

Supplementary Fig. S5 Wiseman et al., 2014
