# Supplementary material for: Deregulation of the FOXM1 target gene network and its coregulatory partners in oesophageal adenocarcinoma
Source: Mol Cancer. 2015 Mar 26;14:69. doi: 10.1186/s12943-015-0339-8 (PMC4392876; doi:10.1186/s12943-015-0339-8)
Supplement: Additional file 3: Table S2. — Clinical details of the patient samples. Patients are grouped by tissue type (normal oesophageal tissue and oesophageal adenocarcinoma tissue). Basic demographic details are shown for all patients. Median age and interquartile range (IQR) is shown. Clinical staging and treatment details are provided for the oesophageal adenocarcinoma group. The number of cases is shown with percentages in brackets. Individual T, N, M stage and histologic grade of tumour is shown as well as the overall American Joint Committee on Cancer (AJCC) stage using the 2010 staging criteria. The number of cases with missing data is indicated where necessary. One OAC sample was omitted from the final Nanostring analysis as the gene expression changes showed this to be an outlier. [file 12943_2015_339_MOESM3_ESM.docx]

|  | | **Normal oesophageal tissue** | **Oesophageal adenocarcinoma tissue** |
| --- | --- | --- | --- |
| Number of cases | | 24 | 58 |
| Male | | 7 (29%) | 47 (81%) |
| Age (median, IQR) | | 65.5 (61.5, 75.5) | 72 (63.3, 77) |
| Histologic grade  (differentiation) | Well |  | 2 (3%) |
|  | Moderate |  | 27 (47%) |
|  | Poor |  | 24 (41%) |
|  | Data missing |  | 5 (9%) |
| Depth of invasion | T1 |  | 2 (3%) |
|  | T2 |  | 15 (26%) |
|  | T3 |  | 30 (52%) |
|  | T4 |  | 2 (3%) |
|  | Data Missing |  | 9 (16%) |
| Nodal metastatic disease (N) | Present |  | 38 (66%) |
|  | Absent |  | 18 (31%) |
|  | Data missing |  | 2 (3%) |
| Distant metastatic disease (M) | Present |  | 20 (34%) |
|  | Absent |  | 37 (64%) |
|  | Data missing |  | 1 (2%) |
| AJCC 2010 Stage | 1 |  | 4 (7%) |
|  | 2 |  | 17 (29%) |
|  | 3 |  | 16 (28%) |
|  | 4 |  | 20 (34%) |
|  | Missing |  | 1 (2%) |
| Treatment | Surgery alone |  | 9 (16%) |
|  | Surgery and chemotherapy |  | 16 (28%) |
|  | Radiotherapy alone |  | 5 (9%) |
|  | Chemotherapy alone |  | 4 (7%) |
|  | EMR |  | 1 (2%) |
|  | Palliative |  | 23 (40%) |

**Additional file 2: Table S2. Clinical details of the patient samples.** Patients are grouped by tissue type (normal oesophageal tissue and oesophageal adenocarcinoma tissue). Basic demographic details are shown for all patients. Median age and interquartile range (IQR) is shown. Clinical staging and treatment details are provided for the oesophageal adenocarcinoma group. The number of cases is shown with percentages in brackets. Individual T, N, M stage and histologic grade of tumour is shown as well as the overall American Joint Committee on Cancer (AJCC) stage using the 2010 staging criteria. The number of cases with missing data is indicated where necessary. One OAC sample was omitted from the final Nanostring analysis as the gene expression changes showed this to be an outlier.
